# Role of Glycogen Synthase Kinase-3β in APP Hyperphosphorylation Induced by NMDA Stimulation in Cortical Neurons

**DOI:** 10.3390/ph3010042

**Published:** 2010-01-07

**Authors:** Cristina Ploia, Alessandra Sclip, Alessio Colombo, Mariaelena Repici, Fabrizio Gardoni, Monica Di Luca, Gianluigi Forloni, Xanthi Antoniou, Tiziana Borsello

**Affiliations:** 1 Istituto di ricerche Farmacologiche "Mario Negri", Via La Masa 19, 20156 Milano, Italy; E-Mails: cristina.ploia@marionegri.it (C.P.); alessandra.sclip@marionegri.it (A.S.); alessiovittorio.colombo@marionegri.it (A.C.); gianluigi.forloni@marionegri.it (G.F.); xanthi.antoniou@marionegri.it (X.A.); 2 UMR 7102 Neurobiologie des Processus Adaptatifs, Universite P. et M. Curie, 9 quai St Bernard, 75005, Paris, France; E-Mail: mr200@le.ac.uk (M.R.); 3 Dipartimento Scienze Farmacologiche, Università degli Studi di Milano, Via Balzaretti, 9, 20133 Milano, Italy; E-Mail: fabrizio.gardoni@unimi.it (F.G.); monica.diluca@unimi.it (M.D.L.)

**Keywords:** APP, NMDA, GSK-3β, JNK, Cdk5

## Abstract

The phosphorylation of Amyloid Precursor Protein (APP) at Thr^668^ plays a key role in APP metabolism that is highly relevant to AD. The c-Jun-N-terminal kinase (JNK), glycogen synthase kinase-3β (GSK-3β) and cyclin-dependent kinase 5 (Cdk5) can all be responsible for this phosphorylation. These kinases are activated by excitotoxic stimuli fundamental hallmarks of AD. The exposure of cortical neurons to a high dose of NMDA (100 μM) for 30’-45’ led to an increase of P-APP Thr^668^. During NMDA stimulation APP hyperphosphorylation has to be assigned to GSK-3β activity, since addition of L803-mts, a substrate competitive inhibitor of GSK-3β reduced APP phosphorylation induced by NMDA. On the contrary, inhibition of JNK and Cdk5 with D-JNKI1 and Roscovitine respectively did not prevent NMDA-induced P-APP increase. These data show a tight connection, in excitotoxic conditions, between APP metabolism and the GSK-3β signaling pathway.

## 1. Introduction

Alzheimer’s disease (AD) is the most common form of dementia in the elderly. One of the pathological hallmarks of AD is the abnormal accumulation of amyloid-β (Aβ) produced by proteolytic cleavage of the Amyloid Precursor Protein (APP). APP processing is influenced by post-translational modifications, *N*- and *O*-glycosylations, and phosphorylations that can induce a preferential cleavage of APP in the amyloidogenic pathway eventually leading to Aβ production [1,2]. In particular the hyperphosphorylation of APP at Threonine 668 (Thr^668^) in the cytoplasmic domain plays a pivotal role in APP processing and is highly relevant to AD [3]. 

The candidate kinases, responsible for Thr^668^phosphorylation, are three: Cyclin-dependent kinase 5 (Cdk5), glycogen synthase kinase-3β (GSK-3β) and c-jun-*N*-terminal kinase (JNK) [4,5,6,7], but the conditions and the precise role of these kinases in APP metabolism have still to be elucidated [8]. These three kinases are also associated with neurotoxicity [9,10] and are implicated in Alzheimer’s disease [5,6,11,12].

Amongst them, JNK is a key pathway in excitotoxicity [13,14,15], Aβ toxicity [16] and AD pathology [3] and modulates APP phosphorylation in differentiated neurons [7,17]. Cdk5 is another kinase with an important role in excitotoxicity [18] as well as AD pathogenesis [19]. Cdk5 and its activator p25 accumulate in neurons during oxidative stress and treatment with Aβ [9,10,20], while altered Cdk5/p25 levels have been reported in AD brains [21]. 

Finally, GSK-3β is of particular relevance to neurological disorders since it phosphorylates APP at Thr^668^, as well as Tau and Presenilin1 [22,23,24], inducing amyloid plaques and neurofibrillary tangles formation [25]. An abnormal increase of GSK-3β activity is associated with AD pathogenesis [26] and inhibition of GSK-3β hyperactivation induced by exposure of neurons to Aβ peptide, prevents neurodegeneration [27]. Moreover GSK-3β inhibition reduces neuronal death in models of oxygen-glucose deprivation as well as glutamate excitotoxicity *in vitro* [28].

Because glutamate playsa key role in AD pathology and NMDA stimulation for prolonged periods leads to increased production and secretionof Aβ fragments in primary neuronal cultures [29] we investigated APP phosphorylation at Thr^668^ in cortical neurons stimulated with a high dose of NMDA (100 μM). This dose of NMDA correlated with an increase in APP phosphorylation and amyloidogenic processing (β-APPs) and led to activation of all three kinases without causing neuronal death in this short temporal window. 

## 2. Results and Discussion

### 2.1. NMDA treatment of cortical neurons

**Figure 1:**
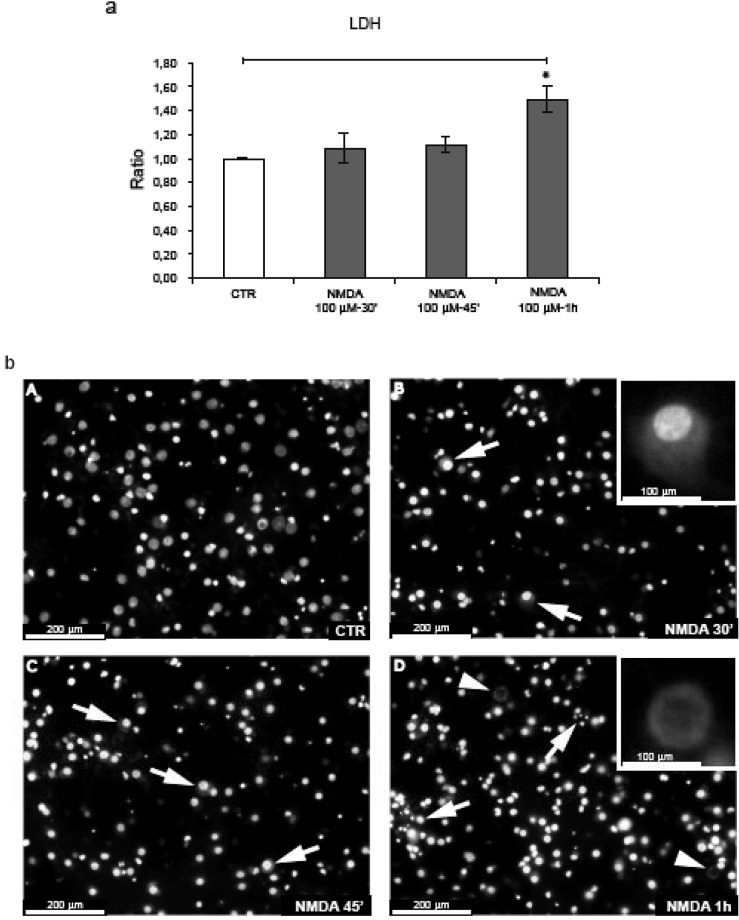
Neuronal death is not induced after 30’-45’ NMDA application. (a) Neurons were exposed to NMDA (30’-45’-1 h) and neuronal viability was assessed by LDH assay. NMDA-1h significantly increased neuronal death. Quantification is from five independent experiments (±S.E.M.), (* p < 0.05). (b) Hoechst staining of neurons exposed to NMDA. At 30’-45’, NMDA induces some neuronal swelling (arrows fig. B-C, magnification 20 × and higher magnification (40 ×) in the box B). After 1 h some neurons undergo nuclear fragmentation (arrows fig. D) while we can also note the presence of the first “ghost” neuron (arrowheads fig. D; higher magnification (40 ×) in the box).

Cortical neurons were exposed to *N*-methyl-D-aspartate (NMDA) 100 μM for 30’-45’. As reported previously by Borsello *et al*., this dose of NMDA induces 90% neuronal death after 24 h [15]. Instead, in the temporal window used in our experiments, NMDA administration did not induce neuronal death as demonstrated by LDH analysis and Hoechst staining (Figure 1, a-b). Following 30’-45’ NMDA stimulation, some neurons presented a swelling appearance with enlarged nuclei, a hallmark of neuronal death (Figure 1b, B-C arrows). 

The first clear signs of neuronal death became apparent after 1 h of NMDA stimulation, with (a) a significant increase in LDH release in comparison to control and NMDA treated cultures for 30’-45’ (b) nuclear fragmentation (Figure 1b, D arrows) and (c) the presence of “ghosts” neurons (denucleated neurons with an unstained shadowy center where the nucleus used to be) (Figure 1b, D arrowheads) further underlying that at this point NMDA becomes toxic. 

### 2.2. NMDA for 30’-45’ induces APP hyperphosphorylation at Thr^668^

**Figure 2:**
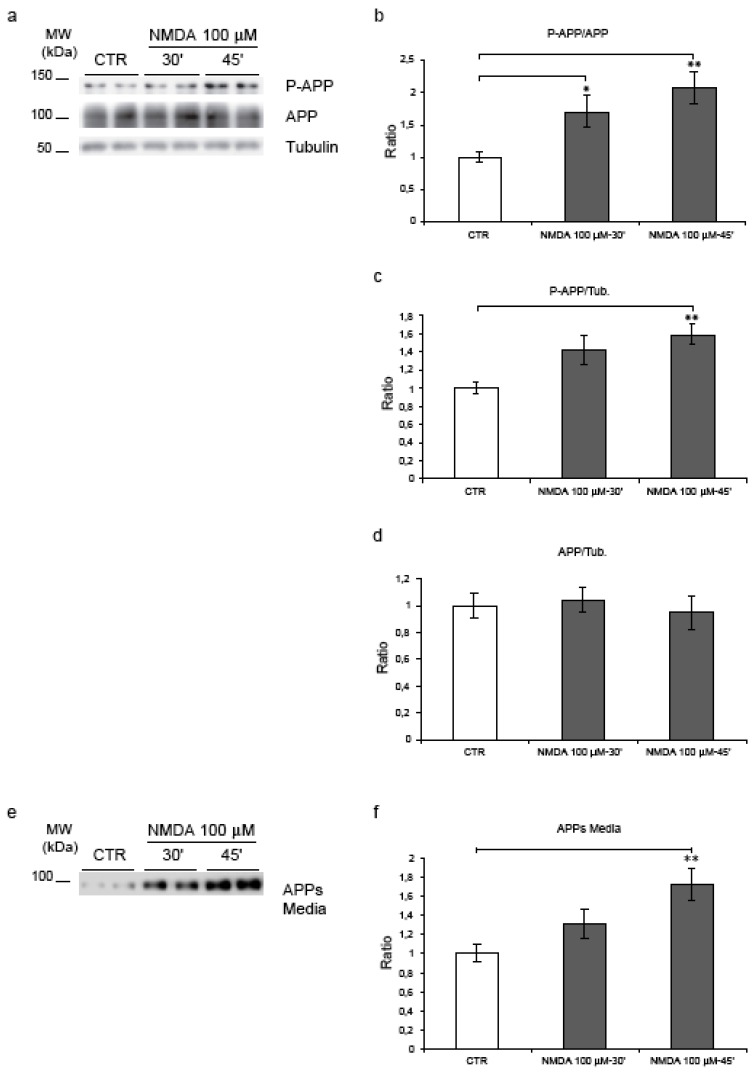
NMDA application induces APP hyperphosphorylation. (a) Neurons were exposed to NMDA (30’-45’), cell lysates were immunoblotted for P-APP and total-APP. Loading control: Tubulin. (b) Quantification showed that NMDA increased P-APP level (P-APP/APP ratio), at 30’ (70%) and 45’ (110%). (c) NMDA increased P-APP/Tub ratio after 45’ (60%). (d) NMDA did not affect the total-APP protein level (APP/Tub). (e) Proteins from culture media were blotted for total-APP secreted (APPs). Loading control: Total medium proteins identified with Coomassie Blue. (f) NMDA-45’ treatment increased APPs level (70%). Quantifications are from six independent experiments (±S.E.M.), * p < 0.05, ** p < 0.01.

Application of 100 μM NMDA in cortical neurons induced hyperphosphorylation of APP at Thr^668^.P-APP was normalized against the total APP using the 22C11 antibody that recognizes the APP full length (Figure 2, a). Quantification of western blots revealed that, compared to control conditions, NMDA application increased APP phosphorylation at Thr^668^. In particular, at 30’ the P-APP/APP ratio reached 1.7 fold increase (p = 0.02) and at 45’ rose further (2.1-fold increase, p = 0.003) (Figure 2b). Total APP protein levels were not affected by NMDA treatment (Figure 2, d). The ratio APP/tubulin did not change, while the ratio P-APP/tubulin increased confirming the augmentation of P-APP (Figure 2, c). Notably, NMDA resulted in an increase of the secreted APPs fragments in the corresponding neuronal media (Figure 2, e). More specifically, by 45’ we observed a 1.7-fold increase (p = 0.003), (Figure 2, f).

**Figure 3:**
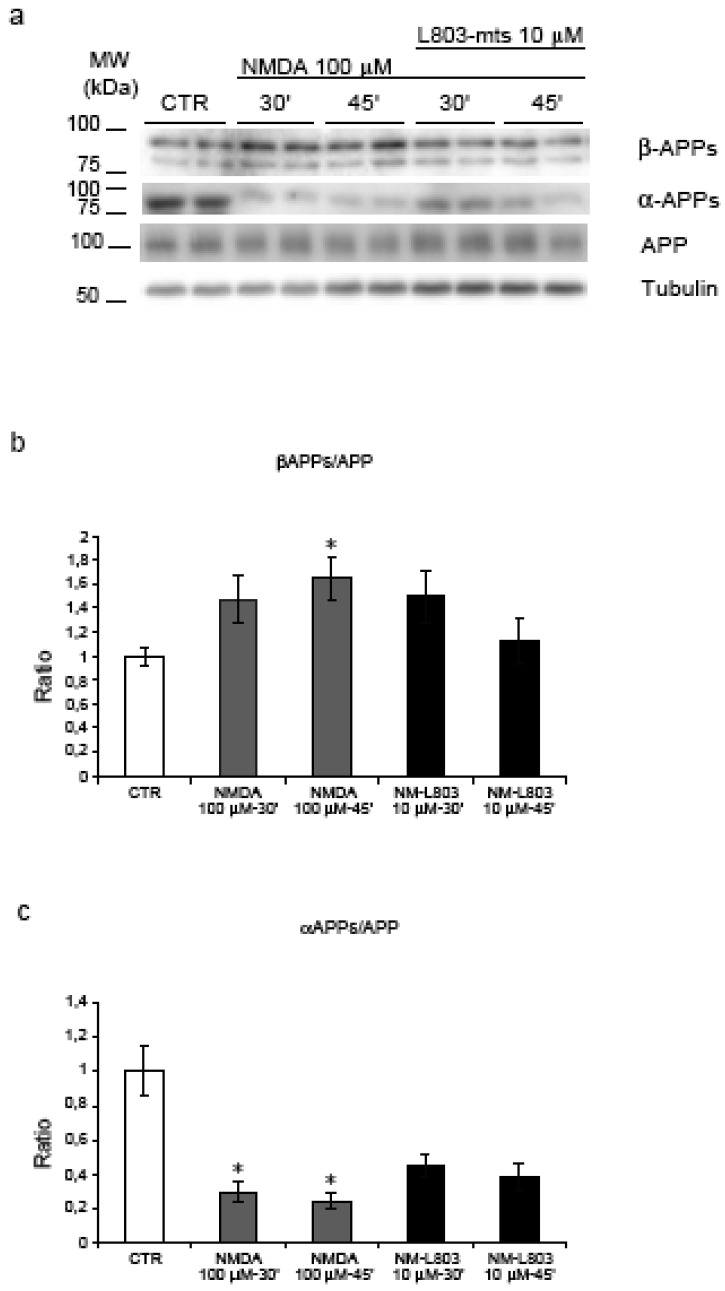
Inhibition of GSK-3β and APP amyloidogenic processing. **(**a) NMDA induces an increase in the APP amyloidogenic processing as shown by the increase of βAPPs and the decrease of αAPPs fragments. (b) Quantification showed a significant increase of βAPPs/APP ratio during NMDA-45’ treatment (p = 0.02) in comparison to untreated controls. L803-mts/NMDA-45’ treatment reduced βAPPs levels, this reduction is not significant compared to NMDA-45’ treatment. **(c) **NMDA reduced the αAPPs/APP ratio at 30’-45’ (p = 0.037, p = 0.039) compared to untreated controls. Instead, L803-mts induced an increase of the αAPPs/APP ratio, although it was not able to restore control levels. Loading control: Tubulin. Quantifications are from three independent experiments (±S.E.M.), * p < 0.05.

Moreover NMDA treatment induced a significant increase in the amyloidogenic processing of APP as demonstrated by the increase of βAPPs/APP ratio after 45’ (p = 0.02) and reduction of αAPPs/APP ratio at 30’-45’ (p = 0.037 and p = 0.039) (see Figure 3, a-b-c). These results suggest that NMDA stimulation, not only induces an increase in APP phosphorylation, but promotes the amyloidogenic processing by increasing βAPPs in the media.

### 2.3. NMDA stimulation induces JNK, Cdk5 and GSK-3β

Because JNK, Cdk5 and GSK-3β kinases can all contribute to APP phosphorylation at Thr^668^ we studied their activation following NMDA stimulation. 

**Figure 4:**
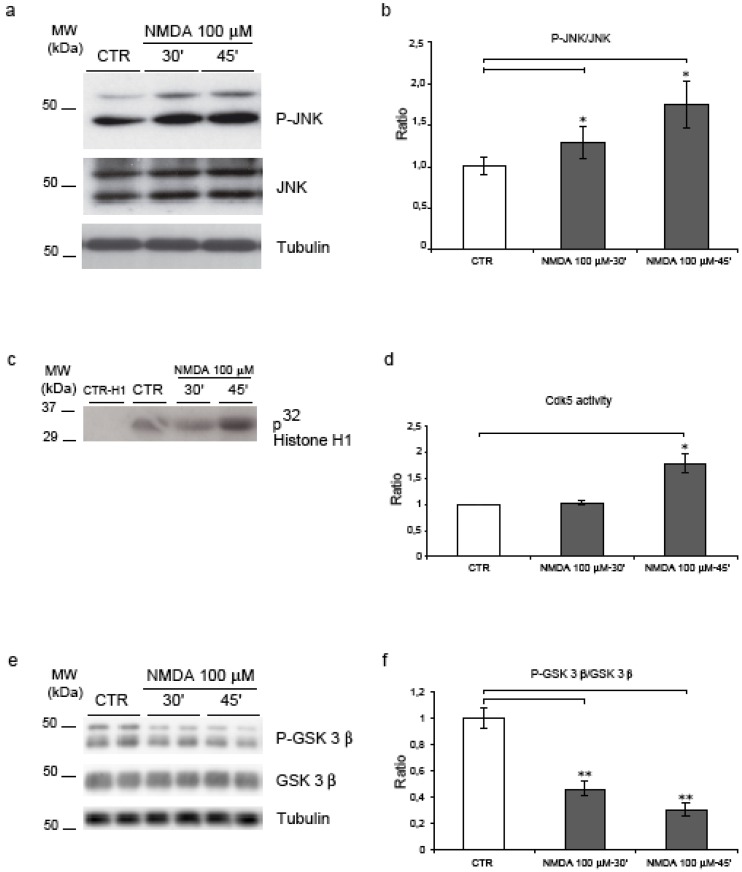
JNK, Cdk5 and GSK-3β are activated following NMDA application. (a) NMDA increased JNK activity as revealed by the increment of P-JNK. (b) Quantification showed a 30% and 75% increase in JNK activity at 30’-45'. (c) Cdk5 activity assay showed an increase in enzyme activity after NMDA-45’. (d) Quantification confirmed an 80% induction of Cdk5 activity after NMDA-45’. (e) Neurons were exposed to NMDA and blotted for P-GSK-3β and total GSK-3β. (f) NMDA induced an increase of GSK-3β activity at 30’ (50%) and 45’ (70%). Loading control: Tubulin. Quantifications are from six independent experiments (±S.E.M.), * p < 0.05, ** p < 0.01.

(1) JNK. In agreement with our previous reports [14,15], we could show that NMDA induces JNK activation both at 30’ and 45’ (1.3- and 1.75-fold, p = 0.03) as shown by quantification of the P-JNK/JNK ratio (Figure 4, a-b). 

(2) Cdk5. The effect of NMDA on Cdk5 activity was examined with Cdk5 immunoprecipitation kinase assays using histone H1 as protein kinase substrate. NMDA application for 45’ induced a significant increase (1.8-fold, p = 0.05) in Cdk5 activity (Figure 4, c-d). 

(3) GSK-3β. Concomitantly, NMDA application led to a powerful activation of GSK-3β (here expressed as a decrease of the inhibitory phosphorylation in Ser-9). GSK-3β phosphorylation decreased to 0.5 (p = 0.002) by 30’, to reach a 0.7 value (p = 0.0002) by 45’ (Figure 4, e-f). 

These results demonstrate that NMDA treatment leads to the activation of all three kinases, indicating the involvement of complex signaling mechanisms.

### 2.4. Neither JNK nor Cdk5 are responsible for NMDA induced APP hyperphosphorylation

**Figure 5:**
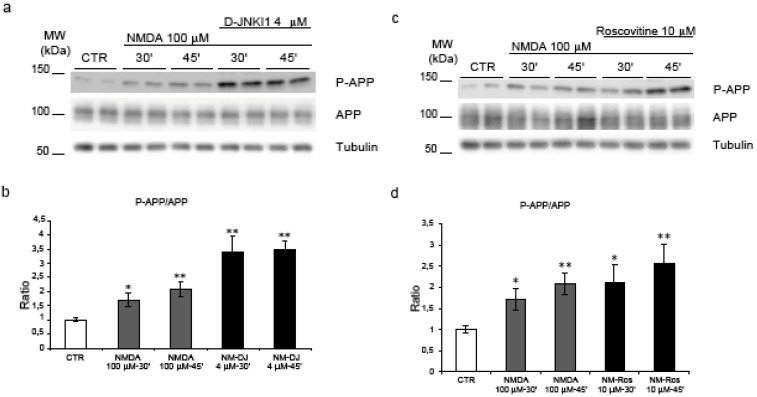
JNK and Cdk5 inhibition do not reverse NMDA induced APP hyperphosphorylation. (a) Neurons were pre-treated with D-JNKI1 and then exposed to NMDA (30’-45’). (b) Western blot analysis and quantification showed that co-treatment with D-JNKI1/NMDA did not reduce P-APP increment induced by NMDA alone. On the contrary, D-JNKI1 pre-treatment increased P-APP/APP level. (NM = NMDA, DJ = D-JNKI1) **(c) **Neurons were pre-treated with Roscovitine and then exposed to NMDA. **(d)** Roscovitine did not reduce P-APP following NMDA application. Roscovitine/NMDA co-treatment further increased P-APP/APP levels compared to controls. (Ros = Roscovitine) Loading control: Tubulin. Quantifications are from six independent experiments (±S.E.M.), * p < 0.05, ** p < 0.01.

**Figure 6:**
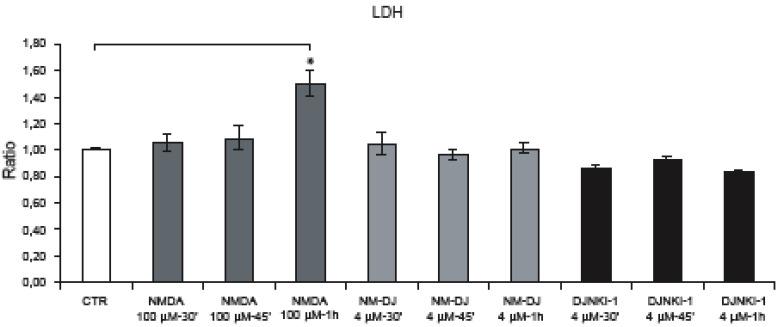
Neurons were exposed to NMDA (30’-45’-1 h) and neuronal viability was assessed by LDH assay. Application of D-JNKI alone or in combination with NMDA (30’-45’) did not affect neuronal survival. NMDA for 1 h significantly increased neuronal death while treatment with D-JNKI protected neurons. Quantification is from five independent experiments (±SEM), (∗ p < 0.05).

As previously shown, in control conditions, D-JNKI1 treatment for 24h prevented APP phosphorylation on Thr^668 ^in cortical neurons [17]. To investigate the role of JNK in APP phosphorylation following NMDA application we used the same D-JNKI1 inhibitor. Neurons were pre-treated with D-JNKI1 (4 μM) 30’ before NMDA stimulation and the P-APP/APP ratio was compared to untreated neurons (Figure 5, a). D-JNKI1 did not prevent NMDA-induced APP phosphorylation as shown by quantification of Western blots (Figure 5, b). On the contrary, D-JNKI1/NMDA co-treatment induced an increase of P-APP compared to NMDA alone. Application of D-JNKI alone or with NMDA did not lead to neuronal death (see Figure 6).

We can conclude that NMDA-induced hyperphosphorylation of APP is not mediated by JNK. 

We then investigated the role of Cdk5 in our model. To prevent Cdk5 action we used the ATP competitive inhibitor Roscovitine, a well-characterized inhibitor of cdc-2 like kinases and the most common inhibitor to block Cdk5 activity [30]. 

Neurons were pre-treated with Roscovitine (10 μM) for 30’ before NMDA administration and the P-APP/APP ratio was compared to untreated neurons. Roscovitine effectively blocked NMDA induced activation of Cdk5 but did not reduce APP phosphorylation levels. Quantification of P-APP/APP ratio confirmed that there was no significant reduction in the Roscovitine/NMDA samples compared to NMDA alone (Figure 5, c-d). 

Thus Cdk5 does not play a pivotal role in NMDA mediated hyperphosphorylation of APP. 

### 2.5. GSK-3β regulates NMDA induced APP hyperphosphorylation

**Figure 7:**
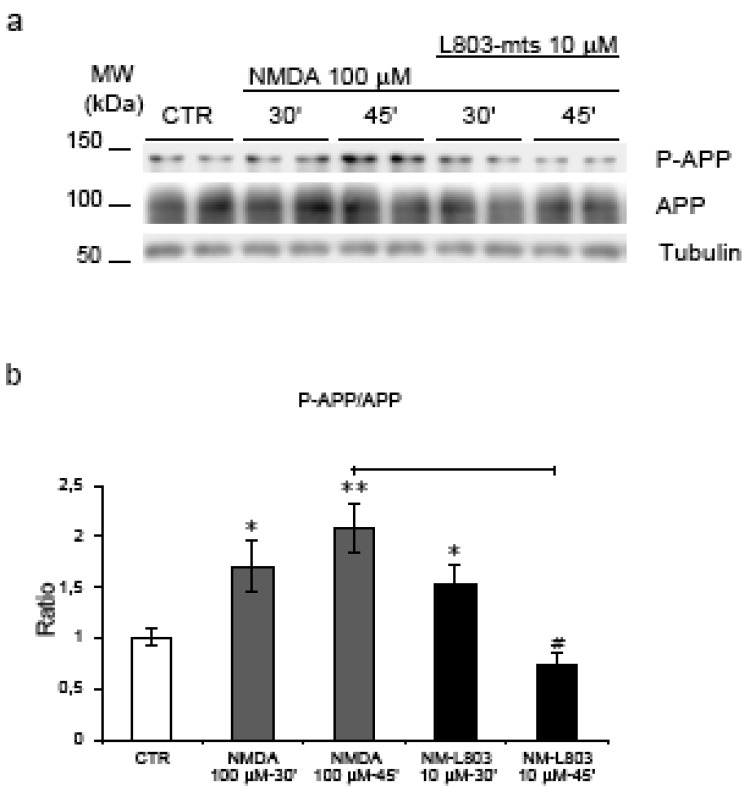
L803-mts action on APP phosphorylation. (a) Neurons were pre-treated with L803-mts and then exposed to NMDA (30’-45’). Western blot analysis shows that L803-mts/NMDA co-treatment inverts the increase of P-APP/APP ratio following NMDA administration. (b) Quantification revealed a significant reduction (65%) of P-APP in L803-mts/NMDA-45’ neurons compared to NMDA-45’(#=p < 0.05). Loading control: Tubulin. Quantifications are from six independent experiments (±S.E.M.), * p < 0.05, ** p < 0.01.

To examine if GSK-3β plays a role in NMDA induced hyperphosphorylation we used L803-mts, a small cell permeable peptide, which competes for the substrate binding site of GSK-3β [31]. Application of L803-mts (10 μM) 30’ before NMDA stimulation led to a reduction of APP phosphorylation during NMDA stimulation at 45’ compared to NMDA alone (p = 0.0006). Notably at 45’, P-APP levels in L803-mts/NMDA treated neurons were comparable to those of non-treated control neurons (Figure 7, a-b) underlying the fundamental role of GSK-3β in this process. We conclude that GSK-3β is the kinase responsible for NMDA mediated hyperphosphorylation of APP.

### 2.6. GSK-3β regulates APP amyloidogenic processing induced by NMDA stimulation

To detect the effect of L803-mts during NMDA treatment on APP amyloidogenic processing we analysed the level of βAPPs (amyloidogenic pathway) and αAPPs fragments (non-amyloidogenic pathway) (Figure 3, a). As described above, NMDA treatment induced a significant increase of βAPPs and a decrease of αAPPs fragments. Following treatment of NMDA stimulated neurons with L803-mts, the βAPPs/APP ratio returned to almost control levels (Figure 3, b), while the αAPPs/APP ratio showed a clear tendency to increase (Figure 3, c). 

These results indicate that L803-mts modulates the APP amyloidogenic processing induced by NMDA.

### 2.7. Discussion

Amyloid Precursor Protein metabolism has a fundamental role in AD pathogenesis. Very important in the processing of APP is the role of the phosphorylation at Thr^668^, in the C-terminal cytoplasmic domain of APP (AICD). Such phosphorylation facilitates β-secretase cleavage and can lead to an increase in Aβ production [3]. It is thus important to elucidate the signaling pathways involved in this phosphorylation and how do they relate to different stressful stimuli.

We investigated the impact of an excitotoxic stimulus such as NMDA on the phosphorylation of APP and on the activation of the three kinases that are involved in this phosphorylation, namely: JNK [6,17], Cdk5 [5,7] and GSK-3β [4]. 

The neurotoxic effect of extracellular aggregates of Aβ peptide, derived from APP processing, is mediated by excitotoxic events such as dysregulation of Ca^2+^ homeostasis, oxidative stress and NMDA responses [32,33,34,35] and recent studies have demonstrated the close interaction between Aβ, Ca^2+^ homeostasis and oxidative stress through the NMDA receptors (NMDA-R) [36,37]. Concomitantly, the NMDA-R has important functions in synaptic transmission, synaptic plasticity and excitotoxicity [38] and deregulation of glutamatergic neurotransmission may contribute to the cognitive deficits present in AD. In fact, memantine, an NMDA receptor antagonist, is a drug used in the treatment of AD and can improve memory in AD patients [39,40]. 

In order to investigate the direct effect of NMDA on APP, we stimulated 12 DIV fully differentiated cortical neurons with NMDA 100 μM for 30’-45’, a combination of concentration and duration that is not toxic for our neuronal *in vitro* model. 

Stimulation of cortical neuronal cultures with NMDA for 30’-45’ induces APP hyperphosphorylation at Thr^668^. Similar data were described by Hoey *et al*., [48]. Additionally, increment in P-APP correlated with an enhanced APP processing as shown by APPs release in neuronal media. Moreover in these conditions we observed an increase of βAPPs and a decrease of αAPPs fragments in neuronal lysates, confirming the important role of APP phosphorylation at Thr^668^ for the APP amyloidogenic processing. 

At the same time, NMDA stimulation for 30’-45’ led to an increase in Cdk5 activity and, in agreement with others, induced an increase of GSK-3β [41] as well as JNK activation [14,15]. It is important to note that APP phosphorylation and activation of these kinases preceded neuronal degeneration, which only became evident after 1 h of NMDA application.

In the second part of this study we investigated the role of Cdk5, GSK-3β and JNK in APP phosphorylation using specific kinase inhibitors. Although several studies have investigated the involvement of these kinases in APP phosphorylation their exact roles in models of excitotoxicity are still very unclear. 

We could show that inhibition of GSK-3β with a substrate competitive inhibitor (L803-mts) abolished NMDA induced APP phosphorylation after 45’ stimulation. Moreover we observed a positive effect of L803-mts on the APP amyloidogenic processing induced by NMDA treatment. In this short time window, the GSK-3β substrate competitive inhibitor led to a decrease of βAPPs production and an increase of αAPPs levels, even if it did not completely restore to control levels. This could be explained by the short-term treatment that is sufficient to significantly reduce P-APP levels but not all the APP processing. 

On the other hand, neither inhibition of Cdk5 by roscovitine, nor inhibition of JNK by D-JNKI reduced NMDA-induced APP hyperphosphorylation. Notably, inhibition of Cdk5 and/or JNK in NMDA stimulated neurons led to a further increase of APP hyperphosphorylation without affecting neuronal survival. Further studies are needed to elucidate the mechanisms behind such regulation. However we think that this data are not so surprising since several studies have reported cross-talks among JNK, GSK-3β and Cdk5 pathways [42,43,44]. Plattner *et al*. described a cross-talk between GSK3 and Cdk5, where Cdk5 over-activation leads to GSK3 inhibition [45]. Similarly, the regulation of GSK-3β activity by JNK was recently demonstrated by Hu *et al*. [46]. We could thus speculate that inhibition of either Cdk5 or JNK could indirectly influence GSK-3 activity and thus APP hyperphosphorylation.

Unfortunately dissecting further these signaling pathways is difficult: a) due to the rapidity of their response and b) because a combinational treatment with these inhibitors was toxic in our model.

## 3. Experimental Section

### 3.1. Cortical Neuronal Culture 

Primary neuronal cultures were obtained from the cerebral cortex of two days post-natal rats, incubated with 200U of papain (Sigma Aldrich) (30’-34 °C), then with trypsin inhibitor (Sigma Aldrich) (45’-34 °C) and subsequently mechanically dissociated. All experimental procedures on animals were performed in accordance with the European Communities Council Directive of 24 November 1986 (86/609/EEC) and all efforts were made to minimise animal suffering. Neurons were plated in 35 mm dishes (~7 × 10^5^ cells/dish) pre-coated with 25 μg/mL poly-D-lysine (Sigma Aldrich). Plating medium was B27/neurobasal supplemented with 0.5 mM glutamine, 100 U/mL penicillin and 100 μg/mL streptomycin. The experiments were performed 12 days from plating date, at which time neurons are considered differentiated. Neurons were treated with NMDA (100 μM, Sigma Aldrich), for 30’-45’-1 h. The inhibitors L803-mts (10 μM, Calbiochem), roscovitine (10 μM, Calbiochem), and D-JNKI1 (4 μM, Xigen SA, Lausanne, Switzerland) were administered to neurons 30’ before NMDA treatment. L803-mts and Roscovitine were diluted in DMSO. Treatment of neurons with DMSO only did not affect the results (data not shown).

### 3.2. Cytotoxicity Assay

Neuronal death was evaluated by a Lactate dehydrogenase assay (LDH), (Cytotox 96 kit, Promega, WI). LDH assays were performed in triplicates.

### 3.3. Cellular Lysis

Total protein extracts were obtained by washing cells twice in ice-cold PBS and lysed (20’-4 °C) in 1% Triton x-100 lysis buffer supplemented with proteases (1 × CPIK, Roche, 10634200) and phosphatases (1 μM 4-NPP, Roche, 10030536) inhibitors [47].

### 3.4. Media Proteins Precipitation

Four hundred μL of medium were incubated with 100 μL of TCA 50% (overnight, 4 °C). After centrifugation (14,000 rpm, 30’-4°C), pellets were washed twice with 500 μL acetone and reconstituted in 50 μL Urea 4 M.

### 3.5. Western Blot Analysis

Protein concentrations were quantified using Bradford Assay (Bio-Rad Protein Assay 500-0006) and 20 μg of whole cell proteins were separated by 8–10% SDS polyacrylamide gel. PVDF membranes were blocked in Tris-buffered saline (5% no fat milk powder, 0.1% Tween20) (1 h, room temperature). Primary antibodies were diluted in the same buffer (incubation overnight, 4 °C) using: 1:2000 anti APP clone 22C11 (APPs) (Chemicon, MAB348), 1:500 anti P-APP (a generous gift from Prof. P. Davis, Albert Einstein College of Medicine of Yeshiva University, NY, USA), 1:250 anti βAPPs (IBL, 18957), 1:1,000 anti αAPPs clone 6E10 (Signet, 9300), 1:2,000 anti P-GSK3β (Ser9) (Cell Signaling Technology, #9336), 1:2000 anti GSK-3β (27C10) (Cell Signaling Technology, #9315), 1:1,000 anti P-JNK (Cell Signaling Technology, #4671) 1:1000 anti JNK (Cell Signaling Technology, #9252). All blots were normalized to α-tubulin (Santa Cruz Biotechnology, sc-8035) and at least three independent experiments were performed. Western blots were quantified by densitometry using Quantity One software (Biorad).

### 3.6. Cdk5 Kinase Assay

Cells were washed in ice-cold PBS and lysed (10 h - 4 °C) in RIPA buffer with proteases (1x CPIK, Roche, 10634200) and phosphatases (1 μM 4-NPP, Roche, 10030536) inhibitors. Three hundred μg of whole cell protein were incubated with 3 μg of anti Cdk5 (C-8) (Santa Cruz Biotechnology, sc-173) (2 h - 4 °C) and precipitated with Protein A Sepharose CL-4B (GE-Healthcare, 17-0780-01) (1 h - 4 °C). Immunoprecipitated complexes were incubated with 6 μg/μL of Histone H1 and 1 μCi/μL of γ-^32^P-ATP in kinase buffer (10 mM Tris, 1mM DTT, 2 mM EGTA, 10 mM MgCl_2_,20 mM ATP) (30’, room temperature). Quantification of kinase assays was done using Quantity One software (Biorad) and based on at least three independent experiments.

### 3.7. Statistical Analysis

All experiments were repeated using at least three independent culture preparations. Quantitative data were statistically analyzed by paired T-test with two-tailed distribution. A *p* value of < 0.05 was considered significant.

## 4. Conclusions 

Altogether, these findings suggest that in adult differentiated stressed neurons GSK-3β is the kinase responsible for APP phosphorylation in excitotoxic conditions and an appropriate regulation of GSK-3β activity can be useful for modulation of APP processing.
